# Generalised Tonic-Clonic Seizure in Adolescents Following COVID-19 Vaccination: A Case Report on a Mere Co-incidence

**DOI:** 10.7759/cureus.40992

**Published:** 2023-06-26

**Authors:** Suresh Babu Mendu, Prathap Reddy Singavarapu, Venu Kota, Abhiram Reddy Sheri, Rakesh Kotha

**Affiliations:** 1 Pediatrics, Government Medical College Siddipet, Siddipet, IND; 2 Surgery, Government Medical College Siddipet, Siddipet, IND; 3 Neonatology, Osmania Medical College, Hyderabad, IND

**Keywords:** child immunity, generalized tonic clonic seizures, adult immunisation, adverse reactions, covid-19 vaccination

## Abstract

Coronavirus disease 2019 (COVID-19) is a novel infectious disease caused by the severe acute respiratory syndrome (SARS) coronavirus 2 (SARS-CoV-2). This disease caused one of the largest pandemics in human history. During the second COVID-19 surge, the Indian government faced the threat posed by the growing COVID-19 pandemic by informing citizens and encouraging the use of preventive measures such as face masks, hand sanitization, personal protective equipment, quarantines, and vaccination. Vaccination is an effective prophylactic intervention in public health, and COVID-19 vaccines have been developed to achieve immunity against viruses and stop the transmission of infection. However, vaccines have side effects, and by early 2021, many doubts arose regarding COVID-19 vaccinations. Few people were not taking immunization because post-immunization adverse events were reported. We are reporting a case of seizures after immunization with Covaxin.

## Introduction

In India, the coronavirus disease 2019 (COVID-19) vaccination drive was successfully initiated and programmed. Nearly 894 million have been vaccinated with the first dose to date, and among them, 27.7 million adolescents (15-18 years old) have been vaccinated with the first dose [1]. The available vaccines in India are Bharat Biotech Vaccine (BBV152, Covaxin) and AstraZeneca (AZD1222, Covisheild). Despite having protection from infection, protecting people from getting seriously ill, and reducing transmission, certain side effects are associated with vaccines. Local pain or discomfort at the injection site and systematic side effects such as headaches, fevers, myalgia, and fatigue are commonly caused by COVID-19 vaccines. Some studies have shown the occurrence of neurological side effects post-vaccination, from simple headaches to cerebral thrombosis and demyelinating diseases [2]. We are reporting a first case of seizure following the administration of Covaxin.

## Case presentation

A 15-year-old male from rural India was brought to the pediatric emergency department on January 20, 2021, by his mother with chief complaints of seizures followed by loss of consciousness. The seizure episode was sudden in onset and characterized by involuntary contractions of both the upper and lower limbs. It lasted 15-20 minutes, then gradually subsided, and the patient was unconscious for one hour, as stated by his mother. There was no previous history of similar episodes in early childhood. No associated comorbidities or family history were noted. This event occurred six to seven hours after the patient received the first dose of the BBV152 vaccine. On detailed inquiry, his mother said that after receiving the vaccine at his school, he seemed fine and was doing normal daily activities, but then, around 10:00 p.m., a seizure episode was noted, and after some time, he became unconscious and was immediately brought to the hospital. At the hospital, the patient was assessed and treated with IV levetiracetam. After about seven to eight hours, the patient became conscious, developed a mild fever, and could not recall what had happened the previous night.

On systemic examination, total neurological and ophthalmological examinations revealed normal findings. The patient was kept for observation because of the fever, and after running investigations (blood glucose, complete urine examination, electrolytes, renal function test, and liver function test), C-reactive protein showed normal values. A smear for malarial parasites was normal. The patient tested positive for dengue NS1 enzyme-linked immunosorbent assay (ELISA) antigen but clinically did not present with any symptoms. On the fifth day of observation, he presented with a mild fever, which subsided within three days. Neither the patient nor his family members suffered from any neuropsychiatric ailments. A brain MRI and CT revealed no relevant findings. The electroencephalogram (EEG) showed occasional discharges (Table 1).

**Table 1:** Lab reports of the patient during the hospital stay

Parameter	Observed value	Reference range
Random Blood Sugar	80 mg/dl	70-140 mg/dl
Sodium (Na+)	139 mEq/L	136-145mEq/L
Potassium (k+)	4mEq/L	3.5-5.2mEq/L
Chloride (Cl-)	103mEq/L	96-106mEq/L
Magnesium (Mg+2)	2.2mg/dl	1.8-2.6mg/dl
Ionized Calcium (Ca+2)	5mg/dl	4.65-5.25 mg/dl
Blood Urea	5 mg/dl	3.5-20 mg/dl
Serum Creatine	1mg/dl	0.7-1.3mg/dl
C-reactive protein (CRP)	0.5mg/dl	0.3-1.0mg/dL
Smear for Malaria Parasite	Negative	Negative
Dengue NS1 Antigen	Positive	Negative
Total Bilirubin	0.3mg/dl	0-0.8mg/dl
Serum Albumin	4.5g/dl	3.5-5.0g/dl
Alanine Transaminase (ALT)	25U/L	10-130U/L
Aspartate Amino Transferase (AST)	30U/L	10-34U/L
Alkaline Phosphatase (ALP)	35U/L	24-147U/L
Gamma- Glutamyl Transferase (GGT)	12U/L	0-25U/L
CT Brain	Normal Study	Normal Study
MRI Brain	Normal Study	Normal Study
Electroencephalogram (EEG)	Seizure activity	Normal Study

A psychiatric assessment was done to rule out lingering or dissociative convulsions. After nine days of admission, the patient was discharged and prescribed 500 mg of levetiracetam twice daily and 10 mg of clobazam for six months. This adverse event following vaccination has been reported to the regional center. One week after the follow-up, the child was fully recovered.

## Discussion

All COVID-19 vaccines have different mechanisms that create antibodies to spike protein (S). Proteins are immunogenic. BBV152 is an inactivated vaccine that does not cause COVID-19 but stimulates the immune system. Vaccinations can cause seizures with and without a fever. The exact cause of these seizures is not fully understood, but they may be caused by adjuvants, preservatives, or immune-inflammatory reactions. Genetic factors like genes PCDH 19 and SCN1A also play a role in postvaccination seizures [3].

Vaccination was found to be the second-most common cause of fever-induced seizures (FS) [4]. Chronic FS are a risk factor for the development of epilepsy, which accounts for 1.16 sudden deaths per 1,000 people annually [4]. Concerns regarding possible neurological complications caused by AZD1222 and BBV152 have been raised in some studies [5]. Previously reported vaccine-induced seizures were reported in Dravet syndrome, in which it seemed that a vaccination-induced fever could trigger a seizure [6]. 

Seizures following COVID-19 vaccination are rarely reported compared to other vaccines. So far, no official report of a BBV152 vaccine-induced seizure has been documented; however, a focal-onset non-motor seizure following Covishield was reported. The patient in this report had cortical atrophy and mild periventricular leukoaraiosis on the left side, which could lead to focal-onset seizures. In this situation, vaccination may trigger seizures [7]. 

Wolf et al. reported seizures associated with other conditions following AstraZeneca and Moderna vaccinations [8]. Lim reported functional neurological disorders after the Pfizer and Moderna vaccines in young people [9]. A Corona Vac multicentric study done in epileptic children showed vaccine safety [10]. Pandit estimated the prevalence of reported adverse events, including seizures, to be 0.2% and concluded in favour of vaccination in the paediatric population. [11]. A case study from China showed that CoronaVac and BBIBP-CorV were safely given to 44 children with tuberous sclerosis [12]. The Pfizer/BioNTech vaccine is recommended for children older than five in the United States and the United Kingdom. Decreased transmission of infection and reduced severity of symptoms are observed after any COVID-19 vaccination. BBV152 is an inactivated vaccine, and no febrile seizure cases have been reported to date. 

Considering the demographic location of India, a differential diagnosis of dengue fever should be made. The patient tested positive for serum dengue NS1 antigen on day three of his admission and presented with a fever the following day, which was treated symptomatically. In rural areas of Telangana state, dengue illness is endemic. The patient's medical history was not accurate. Due to the sudden temperature rise, dengue usually presents with seizures, and the child also had a fever during the hospital stay. Moreover, this case occurred during the high dengue season, and all these factors favour dengue infection. As a result of this, this adverse effect was reported to the Vaccine Adverse Event Reporting System as a category-five coincidental occurrence.

## Conclusions

Healthcare providers should be aware of vaccine-induced adverse events. A single case of seizures after vaccination cannot be treated as a causative factor for post-vaccination seizures. Awareness, education, data reporting, and proper counselling before vaccination can prevent negative incidents and increase the acceptability of vaccines. According to the above studies, COVID-19 vaccines should be used in children because the benefits outweigh the risks.
